# Lower Metabolic Potential and Impaired Metabolic Flexibility in Human Lymph Node Stromal Cells from Patients with Rheumatoid Arthritis

**DOI:** 10.3390/cells12010001

**Published:** 2022-12-20

**Authors:** Tineke A. de Jong, Johanna F. Semmelink, Simone W. Denis, Janne W. Bolt, Mario Maas, Marleen G. H. van de Sande, Riekelt H. L. Houtkooper, Lisa G. M. van Baarsen

**Affiliations:** 1 Laboratory for Experimental Immunology and Department of Rheumatology & Clinical Immunology, Amsterdam UMC Location University of Amsterdam, 1105AZ Amsterdam, The Netherlands; 2 Amsterdam Institute for Infection and Immunity, Inflammatory Diseases, 1105AZ Amsterdam, The Netherlands; 3 Amsterdam Rheumatology & Immunology Center (ARC), Academic Medical Center, 1105AZ Amsterdam, The Netherlands; 4 Laboratory Genetic Metabolic Diseases, Amsterdam UMC Location University of Amsterdam, 1105AZ Amsterdam, The Netherlands; 5 Department of Radiology, Amsterdam UMC Location University of Amsterdam, 1105AZ Amsterdam, The Netherlands; 6 Amsterdam Movement Sciences, Tissue Function and Regeneration, 1105AZ Amsterdam, The Netherlands; 7 Amsterdam Gastroenterology Endocrinology and Metabolism Institute, 1105AZ Amsterdam, The Netherlands; 8 Amsterdam Cardiovascular Sciences Institute, 1105AZ Amsterdam, The Netherlands; 9 Emma Center for Personalized Medicine, Amsterdam UMC, 1105AZ Amsterdam, The Netherlands

**Keywords:** autoimmunity, RA-risk individuals, lymph node stromal cells (LNSCs), mitochondrial respiration

## Abstract

Cellular metabolism is important for determining cell function and shaping immune responses. Studies have shown a crucial role for stromal cells in steering proper immune responses in the lymph node microenvironment. These lymph node stromal cells (LNSCs) tightly regulate immune tolerance. We hypothesize that malfunctioning LNSCs create a microenvironment in which normal immune responses are not properly controlled, possibly leading to the development of autoimmune diseases such as rheumatoid arthritis (RA). Therefore, we set out to determine their metabolic profile during health and systemic autoimmunity. We included autoantibody positive individuals at risk of developing RA (RA-risk individuals), RA patients and healthy volunteers. All study subjects underwent lymph node biopsy sampling. Mitochondrial function in cultured LNSCs was assessed by quantitative PCR, flow cytometry, Seahorse and oleate oxidation assays. Overall, mitochondrial respiration was lower in RA(-risk) LNSCs compared with healthy LNSCs, while metabolic potential was only lower in RA LNSCs. To maintain basal mitochondrial respiration, all LNSCs were mostly dependent on fatty acid oxidation. However, RA(-risk) LNSCs were also dependent on glutamine oxidation. Finally, we showed that RA LNSCs have impaired metabolic flexibility. Our results show that the metabolic landscape of LNSCs is not only altered during established disease, but partly already in individuals at risk of developing RA. Future studies are needed to investigate the impact of restoring metabolic capacity in LNSC-mediated immunomodulation and disease progression.

## 1. Introduction

Rheumatoid arthritis (RA) is an inflammatory autoimmune disease characterized by infiltration of immune cells into the synovium. RA-specific autoantibodies such as rheumatoid factor (RF) and anti-citrullinated protein antibodies (ACPAs) are a hallmark of RA [1]. These autoantibodies can be present in the blood years before clinical manifestation of disease and depending on their risk profile approximately 30–50% of the autoantibody positive individuals develop RA within 3–4 years [2,3,4]. Of importance, synovial inflammation can be absent during this at-risk phase, which suggests that the infiltration of inflammatory cells into synovial tissue occurs at a later stage [5,6]. Since systemic autoimmunity precedes synovial tissue inflammation, it is hypothesized that other unidentified immune processes, possibly outside the synovium, are altered and contribute to disease development. 

Autoimmunity develops when tolerance mechanisms are not properly controlled and self-reactive lymphocytes are activated in the periphery. Self-reactive lymphocytes can escape central tolerance from the thymus and bone marrow, which should then be contained by peripheral tolerance mechanisms working within secondary lymphoid organs, such as the lymph nodes (LNs). In LNs, the modulation of effective immune responses and the regulation of lymphocyte homeostasis depend on the proper functioning of lymph node stromal cells (LNSCs) which form a complex network and physically construct the LN [7,8,9,10]. By producing chemokines, adhesion molecules and survival factors, they guide and position immune cells within the LN and control lymphocyte activation [11,12,13]. Accordingly, LNSCs are key players in immunity and tolerance, and it is essential that LNSCs function is tightly regulated during inflammation. Studies from both human and mice suggest that improper functioning of LNSCs might lead to altered peripheral tissue-restricted antigen (PTA) expression through loss of *DEAF1* transcription, which can induce autoreactive immune cell activation [14,15]. In diabetes type 1 patients, LNSCs have alternative splicing of *DEAF1* mRNA during disease progression which prevents PTA expression [16]. We have shown earlier that LNSCs from RA patients have an altered epigenetic and transcriptomic profile [17], are less responsive to TLR-3 triggering and produce less chemokines upon stimulation with TNF plus lymphotoxin and cytokines crucial for lymph node homeostasis [18,19]. These studies revealed a crucial role for LNSCs in immune regulation, although most knowledge so far is derived from animal models. 

We hypothesize that the malfunctioning of LNSCs leads to a microenvironment where immune responses are not properly controlled, which may lead to the activation of (autoreactive) lymphocytes and the production of autoantibodies. It has been suggested that metabolic pathways and metabolites are critical for cell homeostasis, activation and differentiation [20,21] and (indirectly) promote the resolution of inflammation [21]. For example, tolerogenic dendritic cells (DCs) prefer mitochondrial oxidative phosphorylation and fatty acid oxidation to maintain immune tolerance, while immunogenic DCs prefer glycolysis to trigger immune activation [22,23]. This indicates that distinct metabolic profiles are instrumental for cell function and differentiation and might be interesting targets to switch immune responses during autoimmunity. Metabolic flexibility thus has an important effect on the outcome of cellular responses and tissue function. As it stands, the metabolic profile of LNSCs and how they impinge on their immunomodulatory function remains unclear. In this study, we investigated the metabolic profile of human LNSCs during health, systemic autoimmunity and RA. 

## 2. Materials and Methods

### 2.1. Study Subjects and Tissue Culture

Individuals with arthralgia and/or a family history of RA who were positive for anti-citrullinated protein antibodies (ACPAs; detected by the anti–cyclic citrullinated peptide [anti–CCP] antibody test (CCPlus anti-cyclic citrullinated peptide 2 ELISA (ULN 25 kAU/L) Eurodiagnostica, Nijmegen, The Netherlands) and without any evidence of arthritis upon examination were included. These individuals were considered to be at risk of developing RA (RA-risk individuals), characterized by the presence of systemic autoimmunity associated with RA, but without clinical arthritis (defined as phase c + d, according to EULAR recommendations) [24,25]. After median follow-up of 20 months (11–46 (IQR)), none of these individuals had developed RA despite the presence of ACPAs. However, we expect that 30% of these individuals will eventually develop arthritis within 3–4 years [2,3,4]. For comparison, we included patients with rheumatoid arthritis (ACR/EULAR 2010 criteria [25] who were all biological naïve, ACPA positive and while two patients had longstanding RA (>8 years), three were recently diagnosed with RA. Finally, age-matched seronegative healthy volunteers without any history of autoimmunity or inflammatory disease and no present or previous use of disease-modifying anti-rheumatic drugs (DMARDs) or biologicals were included. Study subjects were recruited either via the outpatient clinic of the Department of Rheumatology and Clinical Immunology at the Amsterdam UMC, via referral from the rheumatology outpatient clinic of Reade, Amsterdam, or via testing family members of RA patients in the outpatient clinic. LN tissues were collected by ultrasound-guided inguinal LN needle core biopsy as previously described [26]. LNSCs were isolated and expanded in vitro as previously described [18]. Experiments were performed using cultured human LNSCs between passages 3 and 5. The study was performed according to the principles of the Declaration of Helsinki [27], approved by the Institutional Review Board of the Amsterdam UMC and all study subjects gave their written informed consent. Table 1 shows the demographics of the included study subjects.

### 2.2. Seahorse Oxidation Assays

Oxygen consumption rates (OCR) and extracellular acidification rates (ECAR) were monitored real-time using the Seahorse XFe96 analyzer and Mito Stress, Mito Fuel Flex and Substrate Oxidation Stress assays according to the manufacturer’s instructions (all Agilent Technologies, Santa Clara, CA, USA) [28]. For all assays, 6–8 technical replicates were seeded in a XF96-well microplate at a density of 10,000 LNSCs/well in normal cell culture medium and incubated overnight under normal cell culture conditions. Additional details of the Seahorse experiments were previously described [28]. Data were analyzed using Wave (version 2.3.0) and Seahorse XF Mito Stress or Fuel Flex test report generators.

### 2.3. Flow Cytometry

For flow cytometry experiments, cultured LNSCs were stained with MitoSOX Red, MitoTracker Green and Image-iT Tetramethylrhodamine, methyl ester (TMRM) as previously described [28]. In short, LNSCs were collected using TrypLE Select (Gibco) for 7 min at 37 °C and washed in Hank’s buffered saline solution (HBSS, BioWhittaker Lonza, Basel, Switzerland). Next, LNSCs were stained with 5 µM MitoSOX Red, 50 nM MitoTracker Green or 100 nM TMRM (all Thermo Fisher Scientific, Landsmeer, The Netherlands) in HBSS for 15 min at 37 °C/5% CO_2_. Subsequently, LNSCs were washed and collected in PBA buffer (PBS containing 0.5% BSA and 0.01% NaN_3_ (Sigma Aldrich, St. Louis, MO, USA)) and measured on a Spectral Analyzer SP6800 (Sony Biotechnology, Weybridge, UK). Data were analyzed using FlowJo v10.8.1 (TreeStar Inc., Ashland, OR, USA). Stainings were normalized for autofluorescence which was determined based on measurements in unstained cells. 

### 2.4. Beta-Oxidation

The production of radiolabeled H_2_O from [9,10-^3^H(N)]-oleic acid was used as a measure to determine the specific activity of the mitochondrial fatty acid β-oxidation flux in human cultured LNSCs, as described previously [28,29]. Measurements were performed using 50,000 LNSCs/well cultured in triplicate in a 48-well plate at 37 °C/5% CO_2_. Oxidation rates were expressed as nanomoles of fatty acid oxidized per hour per milligram of cellular protein.

### 2.5. Quantitative Real-Time PCR

Total RNA and DNA was isolated using the All Prep mini kit (Qiagen, Venlo, The Netherlands) according to manufacturer’s instructions. cDNA was synthesized from total RNA using the RevertAid H Minus First Strand cDNA Synthesis kit (Thermo Fisher). Real-time quantitative PCR (RT-qPCR) was performed using either fast SYBR^®^ Green PCR master mix (Applied Biosystems, Life Technologies, Zwijndrecht, The Netherlands) combined with in house designed primers (Thermo Fisher) or Taqman^®^ Gene Expression master mix combined with Taqman assays (both Applied Biosystems). The primer sequences and Taqman assays used in this study are described in Table 2. The QuantStudio 3 (Applied Biosystems) was used to measure gene expression levels in LNSCs. Each target gene was normalized by the geometric mean of two reference genes: *RPLP0* and *POLR2G* and inter-plate differences were corrected using an arbitrary calibrator sample. The relative quantity (RQ) was calculated using the standard curve method for SYBR green assays or the delta-delta Ct method for Taqman assays. 

### 2.6. Mitochondrial DNA Quantitation

Total DNA was extracted as described above and mitochondrial DNA levels were determined as previously described [28]. In short, RT-qPCR of DNA was performed using the QuantStudio 3 and the quantification of mitochondrial DNA (mtDNA) content relative to nuclear DNA (nuDNA)was determined using the 2 × 2^∆Ct^ method. The following primers were used: mtDNA (mtTL1) PCR forward, CACCCAAGAACAGGGTTTGT; reverse, TGGCCATGGGTATGTTGTTA; and nuDNA (hB2M) PCR, forward, TGCTGTCTCCATGTTTGATGTATCT; reverse, TCTCTGCTCCCCACCTCTAAGT. Data were analyzed using QuantStudio Design & Analysis Software v.1.4.3.

### 2.7. Statistics 

All data are presented as median with interquartile range (IQR). Differences between study groups were analyzed using a non-parametric Kruskal–Wallis test followed by Dunn’s multiple comparison test, to test each group against the control group or a two-way ANOVA test followed by Dunnett’s multiple comparison test, where appropriate. Statistical analysis was performed using GraphPad Prism software (version 9.1.0, La Jolla, CA, USA). *P*-values < 0.05 were considered statistically significant.

## 3. Results

### 3.1. Lower Mitochondrial Respiration in RA-Risk and RA LNSCs

**Figure 1:**
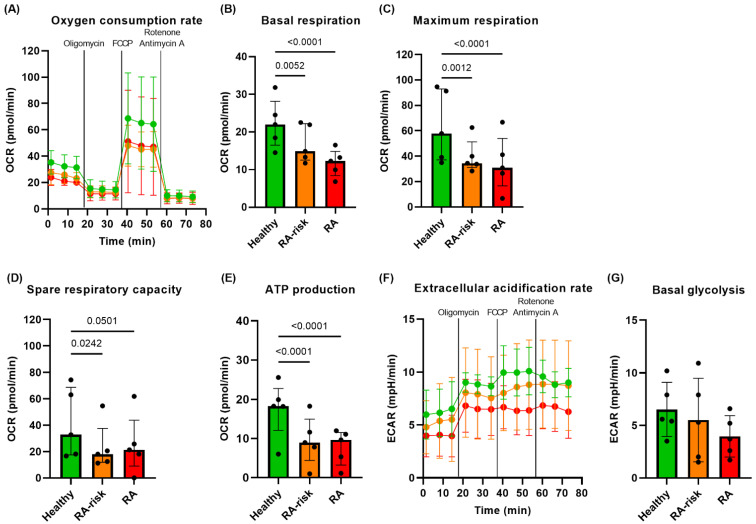
**Lower basal and maximum mitochondrial respiration in RA LNSCs compared with healthy LNSCs.** (**A**) Real-time oxygen consumption rates (OCR) indicating the mitochondrial respiration rate of cultured LNSCs. Basal OCR and extracellular acidification rate (ECAR) measurements were performed before ATP synthase was inhibited using oligomycin to measure the mitochondrial ATP production. After challenging LNSCs with FCCP, maximum mitochondrial respiration and spare respiratory capacity were measured. The addition of rotenone and antimycin A allowed for the estimation of non-mitochondrial respiration rates. (**B**) Basal mitochondrial respiration. (**C**) Maximum mitochondrial respiration. (**D**) spare respiratory capacity. (**E**) ATP production. (**F**) Real-time ECAR indicating the rate of glycolysis of cultured LNSCs. (**G**) Basal ECAR. All donors passage 4, unstimulated conditions. N = 5 per group and five technical replicates per condition, mean value of five replicates per donor is displayed. Data are normalized according to cell input and presented as median + interquartile range. Statistical differences were determined using two-way ANOVA + Dunnett’s T3 multiple comparisons test.

We investigated the metabolic profiles of cultured LNSCs using real-time Seahorse technology. The Seahorse XF96 Analyzer was used to simultaneously measure the oxygen consumption rate (OCR); a measure of mitochondrial respiration or oxidative phosphorylation, as well as the extracellular acidification rate (ECAR); a measure of glycolysis [30]. Overall, RA-risk and RA LNSCs have a lower OCR compared with healthy LNSCs (Figure 1A). Both basal and maximum mitochondrial respiration were significantly lower in RA-risk and RA LNSCs compared with healthy LNSCs (Figure 1B,C). Spare respiratory capacity, an indicator for maximal activation potential, was only significantly lower in RA-risk LNSCs compared with healthy LNSCs (Figure 1D) but inter-individual variability was high. Additionally, mitochondrial ATP production was significantly lower in RA-risk and RA LNSCs compared with healthy LNSCs (Figure 1E). Basal ECAR of cultured LNSCs was not significantly different between donor groups (Figure 1F,G) though variation was high between donors. Overall, mitochondrial respiration levels are lower in RA-risk and RA LNSCs compared with healthy LNSCs, suggesting that metabolic activity of LNSCs is already impaired before the clinical onset of disease. 

### 3.2. Lower Metabolic Potential in RA LNSCs

**Figure 2:**
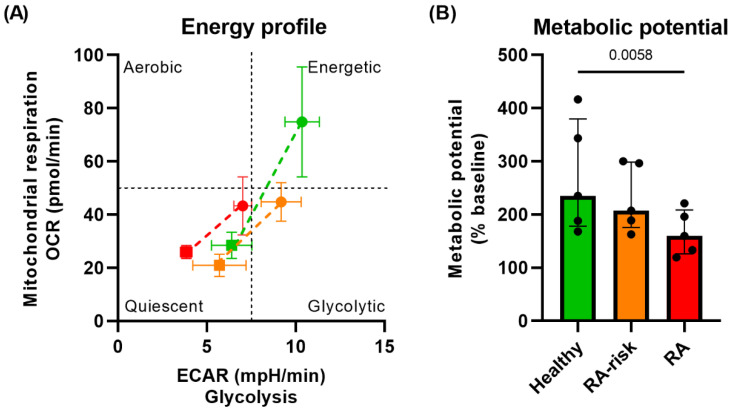
**Lower metabolic potential in RA LNSCs compared with healthy LNSCs.** (**A**) Energy profile of cultured LNSCs at basal mitochondrial respiration (squares) and maximum mitochondrial respiration (circles) based on the OCR and ECAR. (**B**) Metabolic potential of cultured LNSCs. All donors passage 4, unstimulated conditions. N = 5 per group and five technical replicates per condition, mean value of five replicates per donor is displayed. Data are normalized according to cell input and presented as median + interquartile range. Statistical differences were determined using two-way ANOVA + Dunnett’s T3 multiple comparisons test.

Based on the OCR and ECAR levels measured in the Seahorse Mito Stress test, the metabolic potential and energy profile of cultured LNSCs can be determined. The energy profile gives an indication of whether cells are metabolically energetic or more quiescent. During basal mitochondrial respiration, all LNSCs are relatively quiescent (Figure 2A). However, upon maximal mitochondrial respiration activation with FCCP, healthy LNSCs become increasingly more energetic compared with RA-risk and RA LNSCs (Figure 2A). The metabolic potential indicates the fitness of a cell to support a shift from quiescent to energetic and combines the energy profiles at basal and maximum mitochondrial respiration. The resulting quantified metabolic potential is significantly lower in RA LNSCs compared with healthy LNSCs, whereas the metabolic potential of RA-risk LNSCs is similar to healthy LNSCs (Figure 2B). This suggests that despite impaired mitochondrial respiration in cultured RA-risk LNSCs, the cells are still flexible enough to respond to mitochondrial stress. 

### 3.3. Increased Mitochondrial Membrane Potential and ROS Production in RA-Risk LNSCs

**Figure 3:**
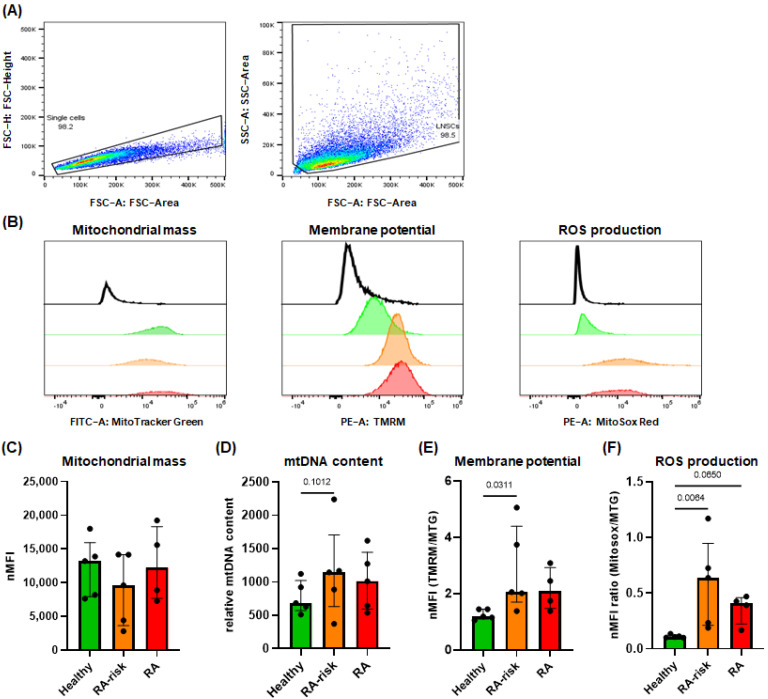
**Higher mitochondrial membrane potential and ROS production in RA-risk LNSCs compared with healthy LNSCs.** (**A**) Flow cytometry gating strategy used to identify LNSCs and single cells. Numbers adjacent to the outlined areas indicate percentages of cells in the gated population. (**B**) Representative histograms of the mitochondrial mass, membrane potential and ROS production of cultured LNSCs. Unstained healthy LNSCs (black), healthy (green), RA-risk individuals (orange) and RA patients (red). MFI quantification was normalized per donor for unstained cells because of inter-individual variation. (**C**) Mitochondrial mass measured using MitoTracker Green. (**D**) Relative mtDNA content normalized against nuclear DNA. (**E**) Mitochondrial membrane potential measured using tetramethylrhodamine, methyl ester (TMRM). (**F**) Mitochondrial ROS production measured using MitoSox Red. All flow cytometry measurements are normalized for unstained cells. Membrane potential and ROS production were additionally normalized for mitochondrial mass. All donors passage 5, unstimulated conditions. N = 5 per group for healthy and RA-risk, N = 4 for RA LNSCs, 2 technical replicates for mtDNA content measurements. Data are presented as median + interquartile range. Statistical differences were determined using Kruskal-Wallis + Dunn’s multiple comparisons test.

Mitochondria are the most important drivers in cellular metabolism. To determine which mitochondrial characteristics are responsible for the lower mitochondrial respiration rates detected in RA-risk and RA LNSCs, we measured the mitochondrial mass, mitochondrial DNA (mtDNA) content, membrane potential and ROS production. The flow cytometry gating strategy used to identify LNSCs and single cells is shown in Figure 3A. Mitochondrial mass as determined using the MitoTracker Green dye and relative mtDNA content were comparable between donor groups, though variation is high within groups (Figure 3B–D). Strikingly, the mitochondrial membrane potential (Figure 3B,E) and ROS production (Figure 3B,F) were significantly higher in RA-risk LNSCs. ROS production in RA LNSCs was numerically, but not statistically significantly, different from healthy LNSCs (Figure 3F). 

### 3.4. Fatty Acid β-Oxidation in Cultured LNSCs

**Figure 4:**
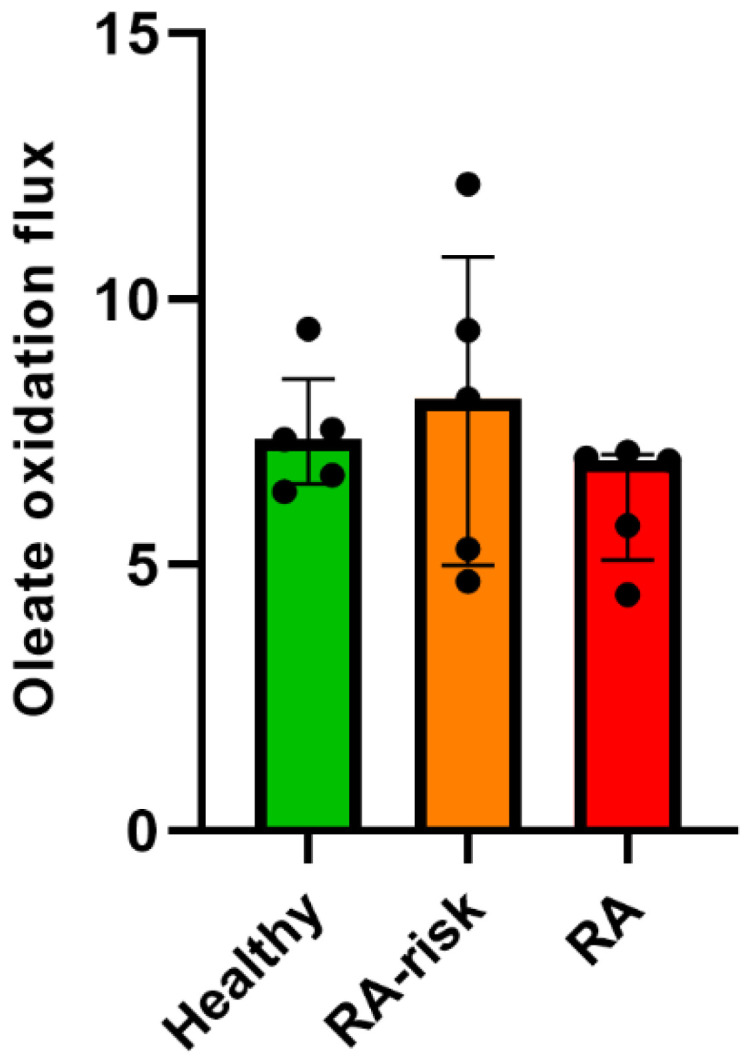
**No significant alteration of fatty acid β-oxidation in cultured LNSCs.** Specific activity (SA) of the oleate flux through the mitochondrial β-oxidation system in nmol per hour per mg protein. All donors passage 3, n = 5 per group and three technical replicates per condition, mean value of three replicates is displayed. Data are presented as median + interquartile range. Statistical differences were determined using two-way ANOVA + Dunnett’s T3 multiple comparisons test.

Lipids are important structural components of the cell membrane. In addition to their structural properties, lipids are used to generate energy through the process of mitochondrial long-chain fatty acid β-oxidation [31]. Interestingly, lymph nodes are surrounded by adipose tissue which facilitates access of the immune system to lipids [32]. A previous transcriptomic analysis of LNSCs showed that genes from the lipid and lipoprotein metabolism pathway were differentially expressed between RA-risk and RA versus healthy LNSCs [17]. We hence proposed that impaired β-oxidation in cultured RA(-risk) LNSCs underlies the observed metabolic changes and determined the β-oxidation flux by the production of radiolabeled H_2_O from a radiolabeled long-chain fatty acid, i.e., [9,10-^3^H(N)]-oleic acid. However, we did not detect any significant differences in long-chain fatty acid β-oxidation between donor groups (Figure 4).

### 3.5. Altered Substrate Use in RA LNSCs

**Figure 5:**
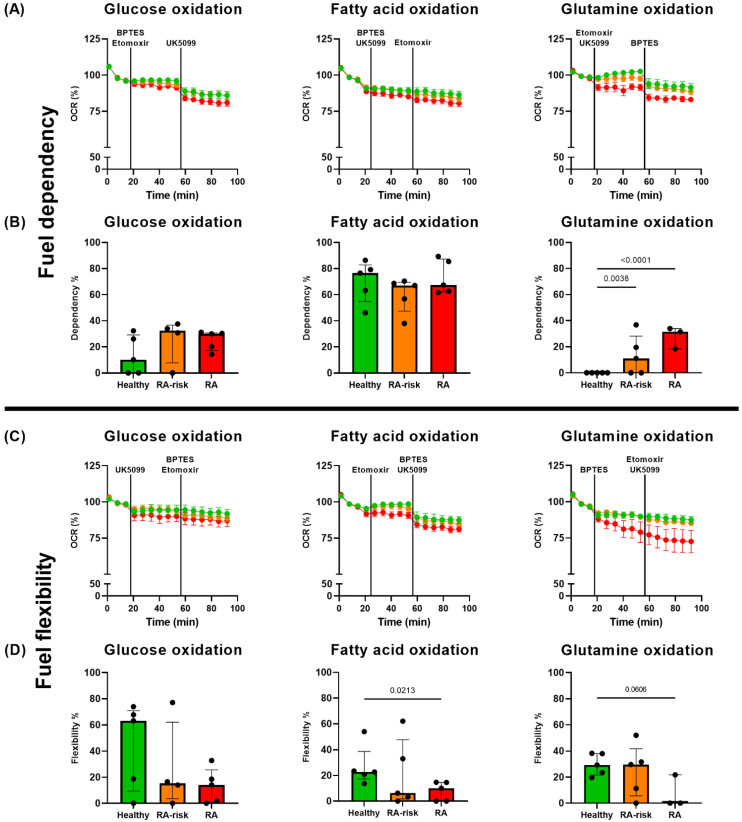
**RA LNSCs are highly dependent on glutamine metabolism and not flexible to switch metabolic pathways compared with RA-risk and healthy LNSCs.** (**A**) Real-time Seahorse measurement of the dependency of cultured LNSCs on glucose, fatty acids and glutamine. (**B**) Dependency of cultured LNSCs on metabolic pathways. (**C**) Real-time Seahorse measurement of the flexibility of cultured LNSCs on glucose, fatty acids and glutamine. (**D**) Flexibility of cultured LNSCs to use a metabolic pathway when the other two pathways are inhibited. All donors passage 5, unstimulated conditions. N = 5 for healthy and RA-risk, n = 3 for RA LNSCs and five technical replicates per condition, mean value of five replicates per donor is displayed. Data are presented as median + interquartile range. Statistical differences were determined using two-way ANOVA + Dunnett’s T3 multiple comparisons test.

Since long-chain fatty acid β-oxidation was not impaired in cultured RA(-risk) LNSCs, we aimed to explore metabolic substrate used in these cells in more detail. Therefore, a Seahorse Fuel Flex assay was used which measures the use and preference of three main metabolic pathways sustaining basal mitochondrial respiration. This assay measures the oxidation dependency and flexibility of one of the metabolic substrates glucose, fatty acids or glutamine relative to the other two. Fuel dependency represents the reliance of LNSCs on a metabolic pathway to maintain basal mitochondrial respiration. RA-risk and RA LNSCs were non-significantly more dependence on glucose oxidation compared with healthy LNSCs (Figure 5A,B). Fatty acid oxidation is relatively high in all donor groups compared with the other two metabolic pathways (Figure 5B). Interestingly, RA(-risk) LNSCs are significantly more dependent on glutamine oxidation compared with healthy LNSCs (Figure 5B). This suggests that LNSCs undergo a metabolic switch during disease development, which already starts before the onset of clinical disease. As fuel availability may change upon inflammatory triggers, we also measured fuel flexibility. This represents how flexible cultured LNSCs are to increase the oxidation of one metabolic pathway to compensate for the inhibition of the other pathways. In line with the impaired metabolic potential of RA cells (Figure 2), RA LNSCs are not flexible to switch to another main metabolic pathway compared with healthy LNSCs, whereas RA-risk LNSCs are still flexible, although variation within donor groups is high (Figure 5C,D). 

In addition to the Fuel Flex assay, we performed so-called Substrate Oxidation Stress assays which give an indication of fuel use during homeostasis and mitochondrial stress. Glucose, fatty acids and glutamine oxidation were individually inhibited to measure relative substrate demand of cultured LNSCs sustaining basal and maximum mitochondrial respiration. These measurements showed that basal mitochondrial respiration was significantly lower when glutamine oxidation was inhibited in healthy and RA LNSCs (Supplementary Figure S1A), indicating that cultured LNSCs are dependent on glutamine oxidation to maintain basal mitochondrial respiration. The effect of pathway inhibition was less pronounced during maximum mitochondrial respiration, although mitochondrial respiration was most affected when glutamine oxidation was inhibited (Supplementary Figure S1B). 

**Figure 6:**
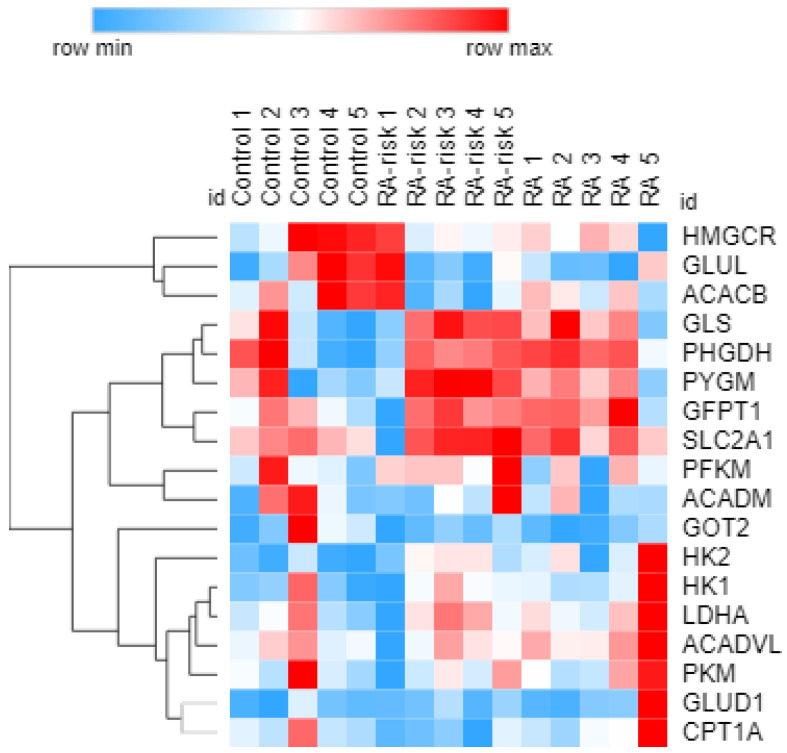
**Gene expression analysis of metabolic pathways in LNSCs.** Hierarchical cluster diagram visualizing the relative expression levels of 18 genes involved in glucose, fatty acid and glutamine metabolism measured using real-time qPCR. Hierarchical clustering was performed using average linking clustering of the rows (genes) only, with metric one-minus spearman rank correlation for non-parametric data. The cluster diagram was created using Morpheus (https://software.broadinstitute.org/morpheus, accessed on 2 September 2022).

Finally, to investigate if a specific step in one of the main metabolic pathways is impaired and potentially causing the observed differences in the metabolic profile of RA (-risk) LNSCs, we measured mRNA levels of genes related to glucose, fatty acid and glutamine oxidation. However, under homeostatic conditions, no significant differences were found between the donor groups, partly due to high inter-individual variation (Figure 6). 

## 4. Discussion

Cellular metabolism is crucial in modulating cell fate and function, thus also for the proper management of immune responses. Inflammation urges cells to enhance their energy production to sustain proliferation and the production of pro- and anti-inflammatory molecules. This suggests that altered cellular metabolism can contribute to dysfunctional immune responses, which is generally also associated with autoimmunity [33]. Metabolic adaptation thus has an important effect on the outcome of cellular responses. As LNSCs not only provide structure to lymphoid organs but tightly control immune cell activation and inhibition, more insight in their metabolic profile may provide novel targets for immunotherapy in autoimmune diseases such as RA. 

To investigate the metabolic profile of LNSCs during health and autoimmunity we compared LNSCs collected from patients with RA with LNSCs from autoantibody positive individuals at risk of developing RA and seronegative healthy volunteers. Of interest, we found that several metabolic alterations identified in RA LNSCs could also be detected in LNSCs from individuals at risk of developing RA.

Metabolic alterations related to oxidative phosphorylation, such as basal and maximum mitochondrial respiration and ATP production, were already significantly lower in RA-risk LNSCs compared with healthy LNSCs and did not further decrease in RA LNSCs, suggesting these metabolic alterations take place before onset of clinical disease. We assume that the proliferation of LNSCs did not influence the detected metabolic alterations, since it has been shown that the proliferation phase of cultured LNSCs starts approximately 27 h after seeding [17]. Although most mitochondrial respiration rates were significantly lower in RA-risk LNSCs, their metabolic potential was still similar to healthy LNSCs. Metabolic potential or flexibility is intimately linked to cellular fitness and is crucial for supporting the required shift from quiescent to active immune cells in case of danger signals [34]. This indicates that RA-risk LNSCs are still able to respond to changing energy demands to regulate immune responses. The observed lower metabolic potential observed in RA LNSCs might indicate that the stromal network in lymph nodes of RA patients is less capable of reacting to surrounding signals to enable proper control of immune responses, potentially leading to decreased immune tolerance. Indeed, our previous studies showed that RA LNSCs are less responsive to TLR-3 triggering and produce less chemokines upon in vitro stimulation [18,19].

Ensuing the distinct differences found in mitochondrial respiration, we investigated whether mitochondrial characteristics such as mitochondrial mass, membrane potential, ROS production and mtDNA content are linked to these differences. The mitochondrial membrane potential has a window in which effects are desirable for providing energy reserves, as low mitochondrial membrane potential might reflect mitochondrial stress and lead to apoptosis, whereas high mitochondrial membrane potential might lead to excessive ROS production through the mitochondrial respiratory chain [35]. We found significantly higher membrane potential and ROS production in RA-risk LNSCs compared with healthy LNSCs, which suggests that the mitochondrial membrane potential of RA-risk LNSCs is outside the window for optimal bioenergetic functionality of mitochondria. Elevated ROS levels can impair ATP synthesis, which suggests that dysfunctional mitochondria might be responsible for the detected decrease in ATP synthesis in RA-risk LNSCs. Furthermore, high ROS levels can impair metabolic pathway function and contribute to inflammation by activating signaling pathways leading to accelerated cellular aging [36], a process which is reported in lymphocytes of patients with established RA [37]. 

Metabolic pathway oxidation assessment showed that compared to glucose and glutamine, LNSCs, similarly to tolerogenic DCs [22,23], are highly dependent on fatty acid oxidation to maintain basal mitochondrial respiration. Surprisingly, healthy LNSCs were hardly dependent on glutamine oxidation, whereas RA LNSCs were significantly more dependent on glutamine oxidation. This implicates a metabolic switch in pathway oxidation during disease development. In line with this, it has previously been shown that other cell types involved in the pathogenesis of RA, such as macrophages, synovial fibroblasts and T cells, also undergo a metabolic switch during disease development [38,39]. Although healthy LNSCs were not very dependent on glucose and glutamine oxidation to maintain basal mitochondrial respiration, they were able to upregulate the oxidation of these pathways when the other two pathways were inhibited. This indicates that healthy LNSCs are metabolically flexible. In contrast, RA LNSCs were not flexible in increasing the oxidation of either pathway when the others are inhibited. Their metabolic inflexibility might be related to the high oxidation rates to maintain basal respiration, causing the pathway to operate at maximal capacity and precluding a further increase in oxidation when the other pathways are inhibited. This also implies that RA LNSCs need all three pathways to function properly. 

Even though the real-time Seahorse measurements show a noticeable drop in total OCR after each of these three metabolic pathways is individually inhibited, not all mitochondrial respiration is inhibited. This might indicate that other metabolic processes contribute to basal and maximum mitochondrial respiration of cultured LNSCs. Other metabolic processes contributing to mitochondrial respiration could be, for instance, short- and medium-chain fatty acids, which do not rely on the carnitine shuttle that is inhibited by etomoxir, or amino acids other than glutamine. Additionally, non-mitochondrial oxygen consumption by other biochemical processes in LNSCs such as very long-chain fatty acids that are oxidized in peroxisomes and other cellular enzymatic processes that consume oxygen could contribute to basal respiration.

To our knowledge, this study is the first to investigate mitochondrial function in cultured human LNSCs during health and disease. Unfortunately, the relatively low number of study subjects included in this study makes it hard to reach statistical significance. However, these unique human lymph node biopsy studies in the context of autoimmunity are extremely challenging to collect and analyze. Moreover, it is challenging to find seronegative healthy volunteers who would like to participate in such a study and LNSCs are typically slow growing cells, thus making these experiments quite labor intensive and time consuming. A limitation of our study setup is that we cannot inhibit all cellular metabolic pathways in parallel, so it is difficult to define the specific contribution of all active metabolic pathways to mitochondrial respiration.

In conclusion, our results show that the metabolic landscape in LNSCs is already altered in autoantibody positive individuals at risk of developing RA, providing an interesting target for future therapies aimed at disease prevention. Mitochondrial dysfunction of LNSCs could disrupt homeostasis and induce loss of tolerance, chronic inflammation and ultimately joint damage. Future studies are needed to investigate how these metabolic alterations of the local stromal microenvironment regulate the immune response in the context of autoimmunity. 

## Figures and Tables

**Table 1:** Demographic baseline characteristics of study subjects.

	Healthy n = 5	RA-Risk Individuals n = 5	RA Patients n = 5
Sex (female, n) (%)	3 (60)	4 (80)	4 (80)
Age (years)	47 (33–58)	48 (36–50)	42 (29–44)
IgM-RF positive (n) (%)	0 (0)	0 (0)	4 (80)
ACPA positive (n) (%)	0 (0)	5 (100)	5 (100)
ESR (mm/h)	ND	5 (2–8.5)	26 (6.5–40.5)
CRP	0.5 (0.3–3.9)	1.6 (0.8–3.6)	3 (1.7–26.5)
Treatment (n) (%)			
NSAIDs	-	-	2 (40)
DMARD	-	-	2 (40)

ND, not determined; IgM-RF, IgM rheumatoid factor; ACPA, anti-citrullinated protein antibodies; ESR, erythrocyte sedimentation rate; CRP, C-reactive protein; NSAIDs, non-steroidal anti-inflammatory drugs; DMARD, disease-modifying antirheumatic drugs. Data are expressed as median (interquartile range) unless otherwise indicated. IgM-RF measured using IgM-RF ELISA, ULN 49 IU/mL (Hycor Biomedical, Indianapolis, IN).

**Table 2:** Overview of primer sequences.

SYBR Green			
Gene	mRNA Transcript ID	Forward Sequence	Reverse Sequence
*POLR2G*	NM_002696.3	GAGGTCGTGGATGCTGTTGT	TCTCTGAAGGGATGGAATGTCG
*RPLP0*	NM_001002.4	GCAGCATCTACAACCCTGAAGT	GCAGACAGACACTGGCAACAT
*ACACB*	XM_011538265.2	GCCCCGAGAACCTCAAGAAA	AGAAGCCGCTGTCCATGAAG
*ACADM*	NM_001286044.1	GGAGTTCACCGAACAGCAGA	AGGGGGACTGGATATTCACCA
*ACADVL*	NM_001033859.2	ATGCACATCCTCAACAATGG	GAATTTTCTCCCCAAACTGG
*CPT1A*	NM_001876.4	GCTGACTCGGTACTCTCTGA	GGGTACACGCCAGTGATGAT
*GFPT1*	NM_001244710.2	AGTTGGCACAAGGCGAGGTA	TGCTGTCCACACGAGAGAGA
*GLS*	NM_014905.5	CAGTAGATGGACAGAGGCATTCT	TTAGTCCACTCGGCTCTTTTCC
*GLUD1*	NM_005271.5	AACTACCACTTGCTCATGTCTG	ATTGTGTATGCCAAGCCAGAGT
*PHGDH*	NM_006623.4	AATCTGCGGAAAGTGCTCATC	GGTGGCAGAGCGAACAATAA
*GLUL*	NM_001033044.4	GGGAGGAGAATGGTCTGAAGTACA	CGATTGGCTACACCAGCAGA
*GOT2*	NM_002080.4	ATGGGCTTATATGGTGAGCGT	CGCAAATCTGGGGTGTTCAG
*HK1*	NM_001358263.1	AGAGGACTGGACCGTCTGAA	GCATGATTCTGGAGAAGTGTGG
*HMGCR*	NM_000859.3	TTTGGGTATTGCTGGCCTTT	TCCCTTACTTCATCCTGTGAGT
*LDHA*	NM_005566.4	CAGGTGGTTGAGAGTGCTTATG	TGTCCCAAAATGCAAGGAACAC
*PFKM*	NM_000289.6	TGGAGATGCCCAAGGTATGAAT	CACTTCCAATCACCGTGCCT
*PKM*	NM_002654.6	TGGAATGAATGTGGCTCGTCT	GATGGGGTCAGAAGCAAAGC
**Taqman assays**			
**Gene**	**Assay ID**		
*POLR2G*	Hs00275738_m1		
*RPLP0*	Hs00420895_gH		
*HK2*	Hs00606086_m1		
*SLC2A1*	Hs00892681_m1		
*PYGM*	Hs00989942_m1		

## Data Availability

Not applicable.

## Supplementary Materials

The following supporting information can be downloaded at: https://www.mdpi.com/article/10.3390/cells12010001/s1, Figure S1: Mitochondrial respiration is most affected upon inhibition of glutamine oxidation.
