# Supplementary figures and images for: Lower Metabolic Potential and Impaired Metabolic Flexibility in Human Lymph Node Stromal Cells from Patients with Rheumatoid Arthritis
Source: Cells. 2022 Dec 20;12(1):1. doi: 10.3390/cells12010001 (PMC9818527; doi:10.3390/cells12010001)

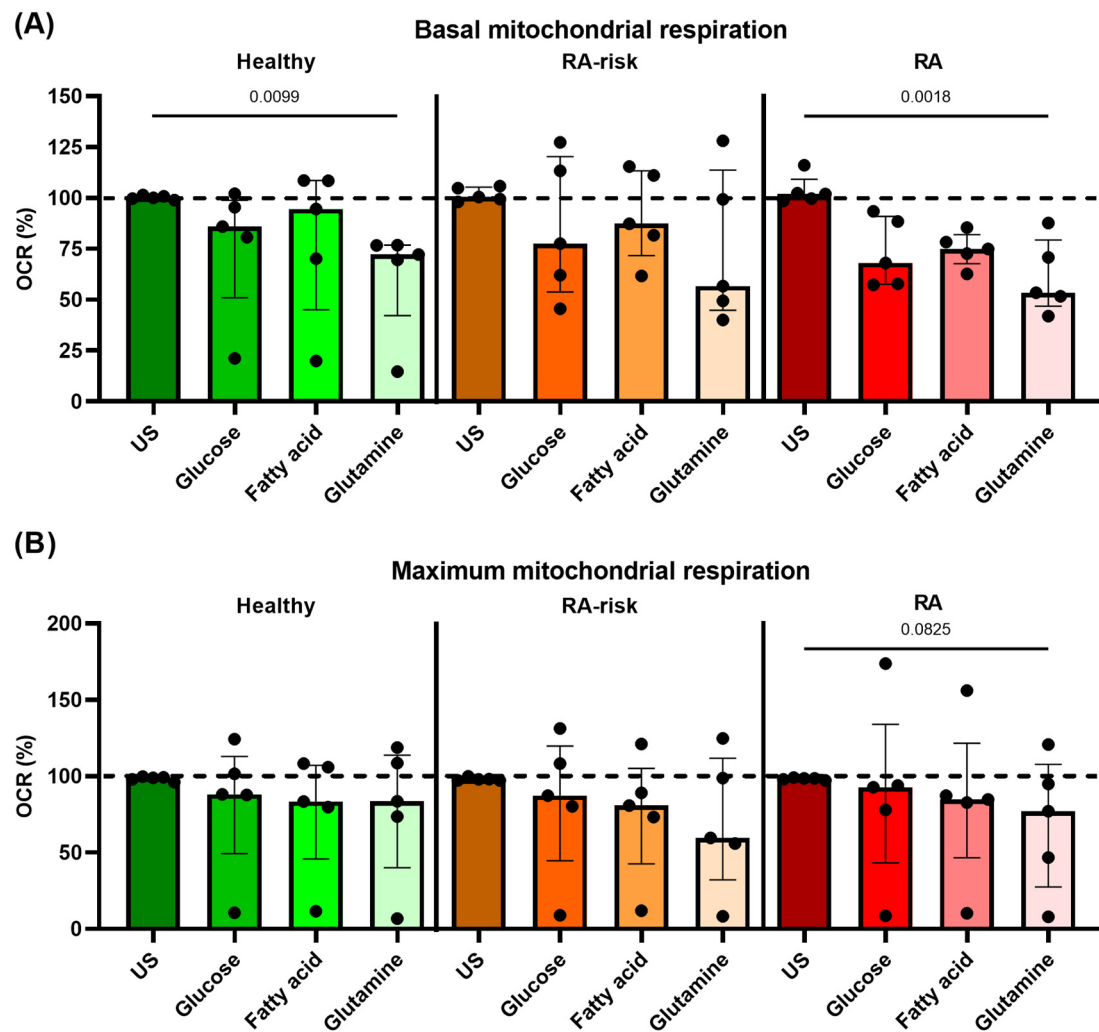

**Figure S1.** Mitochondrial respiration is most affected upon inhibition of glutamine oxidation.

Supplement: Supplementary file 1 [file cells-12-00001-s001.zip › cells-2043774-supplementary.pdf]
